# Usefulness of the Berg Balance Scale for prediction of fall risk in multiple sclerosis

**DOI:** 10.1007/s10072-024-07318-w

**Published:** 2024-01-13

**Authors:** Ender Ayvat, Mert Doğan, Fatma Ayvat, Özge Onursal Kılınç, Gülşah Sütçü, Muhammed Kılınç, Sibel Aksu Yıldırım

**Affiliations:** 1 Department of Neurological Rehabilitation, Faculty of Physical Therapy and Rehabilitation, University of Hacettepe, Ankara, Turkey; 2 Department of Physiotherapy and Rehabilitation, Faculty of Health Sciences, University of Akdeniz, Antalya, Turkey

**Keywords:** Multiple sclerosis, Fall, Berg Balance Scale, Balance, Rehabilitation

## Abstract

**Introduction:**

The Berg Balance Scale, possibly the most widely used balance-related measure, has gained popularity in clinical trials. It provides information about patients’ balance-related abilities and can be used to assess improvement or worsening after rehabilitation. The aim of this study is to determine the cut-off value of the Berg Balance Scale for the fall risk in patients with multiple sclerosis (MS).

**Methods:**

This study was designed as a prospective descriptive trial, and 186 patients with MS were included. Fall history was recorded by interview; balance was assessed using the Berg Balance Scale (BBS).

**Results:**

The mean ages of 96 patients with a fall history within the previous month and 90 patients without a fall history were 35.98 ± 8.58 and 35.71 ± 9.33 years, respectively. The mean value of the BBS score of the faller group was 49.44 ± 5.43 while 52.36 ± 3.53 in non-faller group. The cut-off value of the BBS for fall risk in patients with MS was determined as 50.50 points.

**Conclusions:**

For patient safety and the success of rehabilitation, it is crucial to evaluate the risk of falling in patients with MS, one of the neurological patient groups where complaints about falling are most prevalent. The results showed that BBS is a sensitive and specific measure for identifying in patients with MS at risk of falling.

## Introduction

The nature of multiple sclerosis (MS) results in a variety of motor, sensory, and/or cognitive deficiencies that affect balance and safe mobility [1]. When compared to healthy controls of similar age, patients with MS experience more falls as a result of these impairments [2]. According to a meta-analysis, 56% of patients with MS will fall at least once per 3 months [3]. Even though the fact that most falls suffered by patients with MS do not result in significant injuries, some occur [2]. It has been documented that falls in patients with MS can result in fractures, cerebral bleeding, and even death [4, 5]. Even when they do not result in major injuries, falls in patients with MS, especially recurrent falls, have an adverse effect on the quality of life by causing social isolation, loss of balance confidence, activity restriction, and other problems [6, 7]. Accurate fall risk measurements are required for clinical practice and research due to the significant prevalence and impact of falls.

Research in this field is gaining popularity due to the high rate of falls among patients with MS and the detrimental effects they are associated with [8]. However, it has been repeatedly noted that research in this area has significant methodological constraints and a lack of consistency, particularly with regard to the definition of a fall, the classification of a faller, and the techniques employed to gather fall data [9, 10]. Even though that a number of scales have been created to evaluate the functional level of patients with MS, the ability to use regularly administered scales in this population of patients also enables comparison of the findings with those from other studies and with diseases [11, 12]. Additionally, raters do not need to receive any special training in order to use commonly used scales. The Berg Balance Scale (BBS), possibly the most widely used balance-related measure, has gained popularity in clinical trials. BBS provides information about patients’ balance-related abilities and can be used to assess improvement or worsening after rehabilitation [11, 13]. The BBS has demonstrated strong inter-rater and intra-rater reliability in people with MS [13, 14]. And the minimal clinically important difference for improvement in balance as measured by the BBS is 3 points, meaning that patients with MS are likely to perceive that as a reproducible and clinically important change in their balance performance [15]. No high-quality study has examined the association between falls and BBS for patients with MS despite the high frequency of falls in this group. The most important goal of physiotherapy and rehabilitation in patients with MS is to minimize the fall frequency and risk of patients and to ensure safe mobility. For this reason, the prediction of the fall risks of the patients plays a key role in the creation of the rehabilitation plan. The aim of the study is to determine the cut-off value of the BBS for the fall risk in patients with MS.

## Materials and methods

### Design and participants

This prospective descriptive study was conducted Faculty of Physical Therapy and Rehabilitation, Hacettepe University. It was approved by the Ethics Committee of the Hacettepe University (GO 22/375).

Inclusion criteria for the study included four parameters:

1. Being diagnosed with multiple sclerosis by a neurologist according to McDonald criteria [16]
2. Being older than 18 years old
3. Having an Expanded Disability Status Scale (EDSS) [17] score ≤ 6 (able to walk independently)
4. Mini-Mental State Examination [18] score ≥ 24 points

Exclusion criteria for the study included three parameters:

1. Patients with a neurological disease other than MS that may affect their ability to walk and stand independently
2. Having pain in the lower extremity joints and a history of fractures in the lower extremities in the last 6 months
3. Patients who do not agree to participate in the study and do not give written consent will be excluded from the study

The patients who agreed to participate in the study were informed in detail about the study. The patients signed the informed consent form that was created in compliance with the advice of the Hacettepe University Ethics Committee.

## Assessments

### Demographics and fall history

The demographics of patients such as age, gender, body weight, height, and disease duration were recorded. Following that, data on patients’ prior fall history (within the previous month) were obtained.

The same therapist (MD) had one-on-one interviews with patients and patients were asked to describe the fall in their own words. The patients were instructed to directly explain the circumstances and consequences (injuries or fear). The patients were classified as a “faller” if they suffered at least one fall in the previous month.

### Berg Balance Scale (BBS)

The BBS is a scale that includes scoring between 0 (not applicable) and 4 (normal performance) for the performance of 14 different tasks. Scores from 0 to 20 indicate a loss of balance, scores from 21 to 40 suggest an acceptable level of balance, and scores from 41 to 56 indicate good balance on this scale, which is examined across 56 points [19].

### Statistical analysis

The SPSS software package (version 20.0, SPSS Inc., Chicago, IL) program was used for statistical analysis. The conformity of the variables to normal distribution was examined by visual (histogram and probability graphics) and by using the Kolmogorov-Smirnov. For numerical variables, means and standard deviations were used. Non-parametric tests were used because the data obtained were not normally distributed. The Mann-Whitney *U* test was used to analyze group differences between patients with and without a history of falls. The capacity of BBS values in predicting falls was analyzed using ROC (receiver operating characteristic) curve analysis. The highest combination of sensitivity and specificity was taken to determine the optimal cut-off (significant limit) value.

## Results

**Fig. 1:**
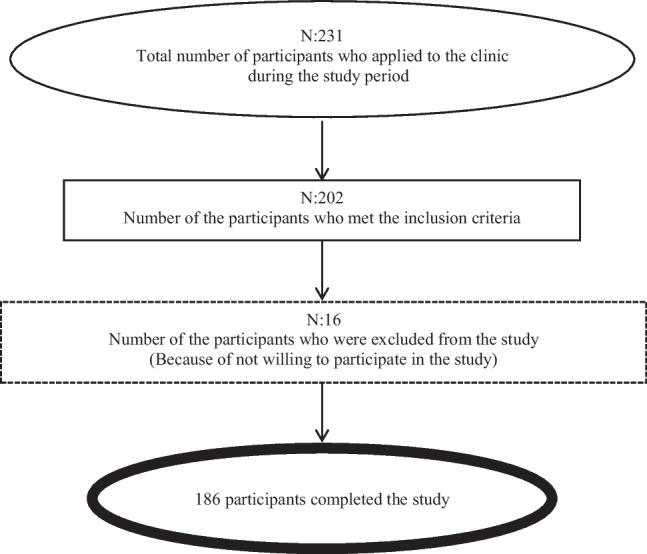
The flow chart of the study

The flow chart of the study is shown in Fig. 1.s Among the 202 patients who met the inclusion criteria, 186 patients who agreed to participate voluntarily were included in the study. One hundred eighty-six patients with MS, consisting of 96 individuals in faller group and 90 individuals in non-faller group, were included in the study. The mean age of the patients were 35.98 ± 8.58 and 35.71 ± 9.33 in faller and non-faller groups, respectively. The demographics and the BBS scores of the individuals are shown in Table 1. The EDSS scores and BBS scores showed a statistically significant difference when the demographic and clinical scores of the two groups were compared (*p* < 0.05). The effect size analysis revealed that the effect of falling on BBS scores was the largest among all.

**Table 1:** Demographics of the patients with MS

	Faller (*n* = 96)		Non-faller (*n* = 90)		*p*
	X ± SD	Median (IQR)	X ± SD	Median (IQR)	
Age (years)	35.98 ± 8.58	37 (13)	35.71 ± 9.33	37 (14)	0.65
Height (cm)	167.21 ± 9.7	164 (15)	165.78 ± 10.81	163 (15)	0.24
Weight (kg)	64.88 ± 14.36	63 (21)	62.46 ± 12.05	62 (17.7)	0.38
BMI (kg/m^2^)	22.68 ± 3.81	22.85 (5)	22.92 ± 3.84	22,36 (5.83)	0.45
EDSS (score)	3.46 ± 1.19	4 (1.5)	3.06 ± 0.88	3 (1.5)	0.02*
Duration of disease (months)	104.67 ± 54.31	96 (69)	109.79 ± 55.58	101 (76)	0.64
BBS (score)	49.44 ± 5.43	50 (6)	52.36 ± 3.53	53.5 (5)	0.001*

*BMI* body mass ındex, *EDSS* Expanded Disability Status Scale, *BBS* Berg Balance Scale, *X* mean, *SD* standard deviation

*p < 0.05 Mann–Whitney *U* Test

**Fig. 2:**
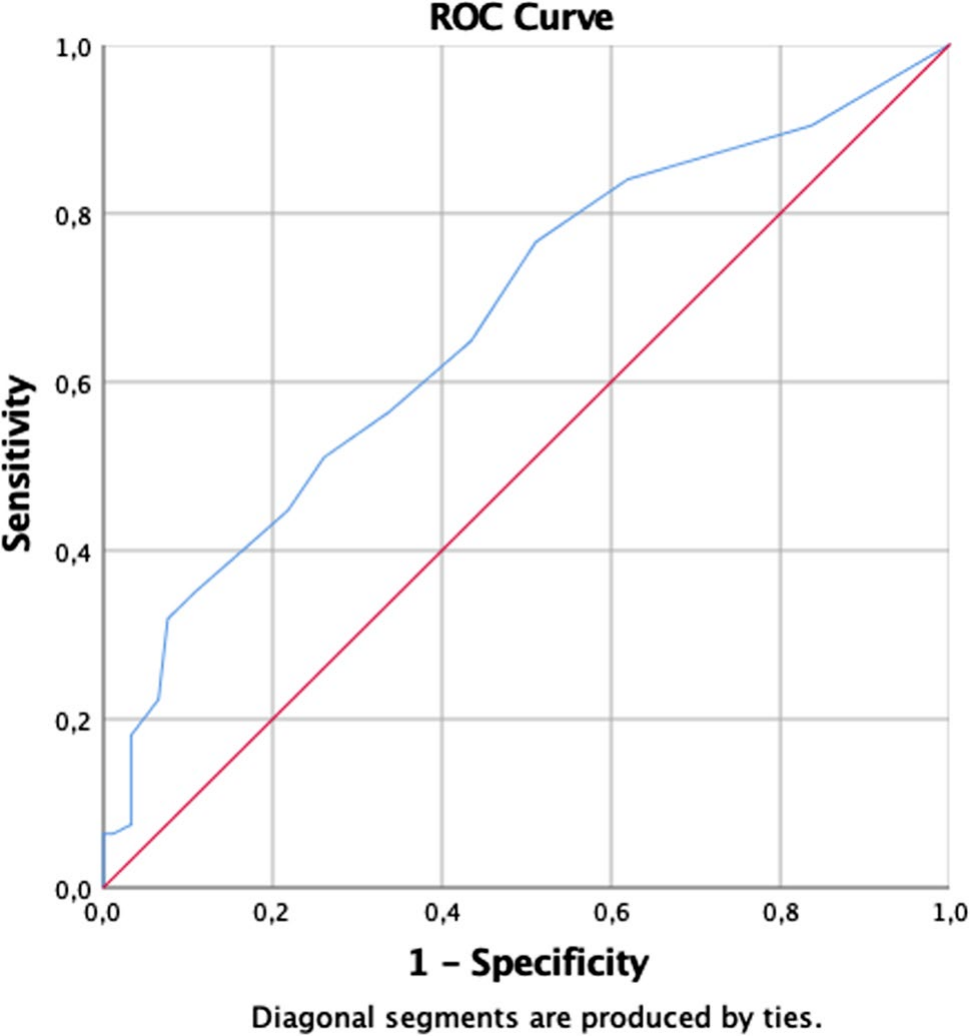
ROC-curve for BBS

According to ROC analysis, the clinical cut-off point for BBS in patients with MS was determined as 50.5 points. Scores that equal to 50.5 points or less with 95% confidence was considered as normal for BBS in patients with MS (95% confidence interval lower bound = 0.596, upper bound = 0.750; area under the curve (AUC) = 0.670; Std error = 0.030; *p* < 0.001) (Fig. 2). The sensitivity and specificity of BBS are shown in Table 2.

**Table 2:** Sensivity and specificity of BBS

	AUC (SEM)	%95 CI	Cut-off point	Sensitivity (%)	Specifity (%)	PPV	NPV	LR +	LR-	DOR
BBS	0.67 (0.03)	0.596–0.75	50.50	51.1	48.9	50.5	47.3	0.95	1.04	0.91

*BBS* Berg Balance Scale, *AUC* area under curve (ROC analysis)

The power analysis of the research was performed with MedCalc software Ltd (Ostend, Belgium). According to priori analysis, the sample size required to reach 95% confidence interval, and 80% power was 87 for AUC value. In the post hoc analysis, the actual power of the research was 98.6% at the 95% confidence interval.

## Discussion

The study aims to determine the cut-off value of the BBS for the fall risk in individuals suffering from MS. The findings of the present study demonstrated a precise BBS measurement to assess the risk of falling in patients with MS, showing that people with scores over 50.5 points have a significant risk of falling. The risk of falling in MS can therefore be assessed using the BBS, a simple and useful balance test.

In this study, the frequency of falls (51.6%) was close but slightly lower concerning to previous findings in the literature (56%); this difference could be due to differences in the characteristics of the sample [3].

Although the fact that studies have shown the BBS to have positive psychometric qualities in patients with MS, there are still some limitations, including ceiling effects, reduced responsiveness, and issues with its rating scale design [11, 20, 21]. Additionally, the BBS was not intended to evaluate several aspects of balance, including responses to external disturbances, dynamic gait, and dual tasks-all of which are crucial for functional balance in patients with MS [22]. Despite such limitations, BBS is one of the most preferred outcome measurements in clinics and researches even advised as “highly recommended” by Neurology Section of the American Physical Therapy Association’s Multiple Sclerosis Taskforce [23].

In order to determine the cut-off value of BBS in the literature, studies have been carried out in different populations, and cut-off values have been revealed with varying scores. In the MS population, the researchers defined two different cut-off values in two separate studies carried out for different aims. Cattaneo et al., in their study aiming to test concurrent and discriminant validity of several balance tests, found the cut-off score of BBS 44 in 51 patients with MS [11]. By Nilsagard et al. discovered the cut-off score as 55 in 76 patients with MS in their study designed to investigate accidental falls and near fall incidents with respect to clinical variables and the predictive values of four tests [24]. Both studies had relatively low sample sizes, and Catttaneo recruited only MS with moderately impaired in balance skills, while Nilsagard recruited only MS with EDSS scores 3.5 to 6. Clinically, it is observed that there is a significant difference between the two cut-off score values. On the other hand, the present study reached a larger sample size (*n* = 186) and found the cut-off value of 50.5 for BBS. In the current study, it was desired to reflect even the MS population more efficiently by including patients with MS in the wider EDSS score range (1–6). We think that the reason for the different cut-offs is the sample size and wider EDDS score range.

The high scores of the patients in the faller group contributed to the test’s low sensitivity. The falls in this category could be the result of various reasons that the BBS test does not account for. Before designing a prospective study, additional characteristics must be taken into account in order to predict falls in this MS population.

The limitation of this study was the functional status of individuals included in the sample group (all participants ambulated independently, with or without aid); although we included a wider EDSS range (1–6) compared to other studies, therefore, the findings may not be generalizable to a wider population of patients with MS. Future studies stratified by the EDSS can be performed to see the behavior of the BSS in different disability strata. In this way, a clearer idea could be obtained with the fall risk of patients at different levels according to the EDSS score. Another limitation of the present study, a history of falls was taken verbally from the patient. Although the history of falls within a period of only a month was taken and the cognition/memory problems of the individuals included were not evident, this may have caused a recall bias. To prevent this, it could be used as a fall log or in different scales.

In conclusion, evaluation and prediction of the risk of falling in patients with MS, one of the neurological patient groups in which fall complaints are most common, is very important for patient safety and rehabilitation success. For this purpose, the BBS can help identify patients who are at risk of falling as a clinical scale that is used widely in patients with MS. However, considering the sensitivity and specificity values of the results of our study, it is recommended to consider alternative scales in cases where a more sensitive assessment of balance is needed.
